# Bioinformatics analysis identifies Mot2 protein as a potential regulator of autophagy in *Saccharomyces cerevisiae*

**DOI:** 10.17912/micropub.biology.001698

**Published:** 2025-07-10

**Authors:** Emmanuella Wesome Avogo, Nicholas A. Burlingame, Swaroopa Badenahalli Narasimhaiah, Elizabeth Delorme-Axford

**Affiliations:** 1 Biological Sciences, Oakland University, Rochester, Michigan, United States

## Abstract

Macroautophagy/autophagy is a conserved mechanism of cellular degradation and recycling. Autophagy is a multi-step process that must be precisely regulated at multiple levels (transcriptional, post-transcriptional, translational, and post-translational). However, there is a gap in our understanding of the molecular details of these mechanisms. Therefore, characterization of factors modulating autophagy and autophagy-related ( *ATG* ) genes is an important area for investigation. Here, we used a bioinformatics approach to screen 37 yeast *ATG* genes using the YEASTRACT database to identify potential regulators. Through our selection criteria, we discovered one novel factor—Mot2p. Our findings support that the yeast Mot2 protein is a regulator of cell survival, *ATG8* , and potentially, autophagy.

**Figure 1. Mot2p is a potential regulator of autophagy identified by YEASTRACT analysis:**
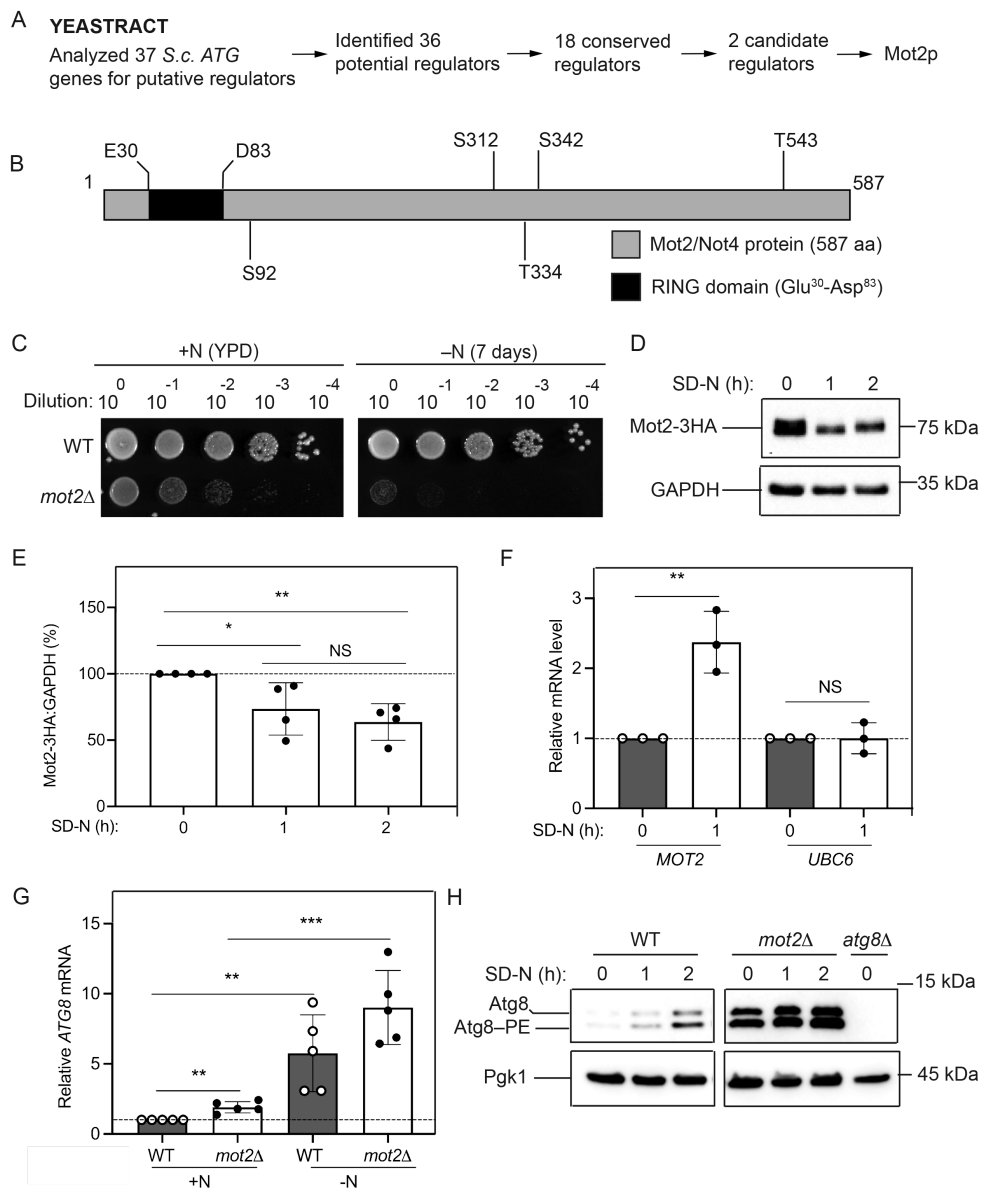
( **A** ) Overview of YEASTRACT approach to identify novel potential regulator(s) of autophagy-related ( *ATG* ) genes and autophagy. ( **B** ) Schematic of the full-length Mot2/Not4 protein, including 587 amino acid residues, RING domain (Glu ^30^ -Asp ^83^ ), and phosphorylation sites (Ser ^92^ , Ser ^312^ , Thr ^334^ , Ser ^342^ , and Thr ^543^ ). ( **C** ) Loss of *MOT2* decreases cell survival after prolonged nitrogen starvation. WT (BY4742) and *mot2* ∆ cells were serially diluted as indicated and grown on YPD plates for 2 days, then imaged. Image is representative of n=3 independent experiments performed in duplicate. ( **D** ) Mot2-3HA fusion protein levels decrease with nitrogen starvation. WT (EWA018) cells endogenously expressing Mot2-3HA fusion protein were grown to mid-log phase in YPD and then starved for nitrogen as indicated. Protein extracts were analyzed by SDS-PAGE and blotted using an antibody that recognizes the HA tag. GAPDH protein is the loading control. A representative blot is shown (n=4). ( **E** ) Densitometry of blots represented in ( **D** ). The percentage of Mot2-3HA:GAPDH was quantified. ( **F** ) *MOT2* mRNA expression increases during nitrogen starvation and autophagy induction. WT (BY4742) cells were grown to mid-log phase in YPD (0 h SD-N) and then nitrogen starved for 1 h. Total RNA was extracted, and RT-qPCR was performed. Results shown are relative to the level of expression in WT cells under rich conditions (+N), which was set to 1 (n=3). ( **G** ) Mot2p negatively regulates *ATG8* mRNA levels. WT (BY4742) and *mot2* ∆ cells were grown to mid-log phase in YPD (+N) and then nitrogen starved (–N) for 1 h. Total RNA was extracted and analyzed as in ( **F** ). Results shown are relative to the level of *ATG8* mRNA expression in WT cells under rich conditions (+N), which was set to 1 (n=5). ( **H** ) Mot2p negatively regulates Atg8 protein levels. WT (BY4742), *mot2* ∆, and *atg8* ∆ (YAB369) strains were assayed for Atg8 protein. Protein extracts were analyzed by urea SDS-PAGE and blotted with anti-Atg8 or anti-Pgk1 (loading control) antibodies. An irrelevant lane was removed from the blot image. A representative blot is shown (n=5). For ( **E** )–( **G** ), results shown are the mean. Error bars indicate standard deviation.

## Description

Canonical macroautophagy/autophagy is a highly conserved mechanism of cellular degradation and recycling. Basal autophagy is essential for homeostasis, but is upregulated under stressful conditions (such as nutrient limitation). Currently, >40 autophagy-related ( *ATG* ) genes have been identified in yeast. This complexity requires strict modulation of the autophagy machinery at multiple levels (transcriptional, post-transcriptional, translational, and post-translational). Despite the need for precision, there is a gap in our understanding of the factors and mechanisms regulating autophagy in the cell. In humans, perturbation of autophagy (i.e., too much or too little) can have deleterious effects on cell health and survival, contributing to disease pathogenesis (Klionsky, Petroni et al. 2021). Therefore, identifying molecular mechanisms modulating *ATG* gene expression and autophagy is an important area for investigation.

The primary goal of this study was to identify and characterize a novel regulator of *ATG* genes and autophagy in the yeast *Saccharomyces cerevisiae* . Using the online database YEASTRACT (Yeast Search for Transcriptional Regulators And Consensus Tracking; www.yeastract.com ) (Teixeira, Monteiro et al. 2006), we screened *ATG1* - *24* , *ATG26* - *27* , *ATG29* , *ATG31* - *34* , *ATG36* , *ATG38* - *42* to identify potential transcriptional regulators ( Figure 1A ). *ATG25* (Monastyrska, Kiel et al. 2005), *ATG28* (Stasyk, Stasyk et al. 2006), *ATG30* (Farré, Manjithaya et al. 2008), *ATG35* (Nazarko, Nazarko et al. 2011), and *ATG37* (Nazarko, Ozeki et al. 2014) are only present in methylotrophic yeast, and no equivalent has been identified in *S. cerevisiae* . YEASTRACT analysis of 37 *S. cerevisiae* *ATG* genes yielded 36 potential transcription factors that were predicted to regulate >50% of the *ATG* genes examined ( Figure 1A ). Next, using the *Saccharomyces Genome Database* ( https://www.yeastgenome.org/ ), we found that 18 of the 36 potential regulators are conserved from yeast to humans ( Figure 1A ). Of the remaining 18, we excluded factors that had published association(s) with autophagy. Of these, we were left with 2 candidates—Spt23p and Mot2p. Spt23p has a human homolog—ANKFY1 *.* ANKFY1 has a published association with autophagy (Park, Peng et al. 2016, Wei, Fu et al. 2024), indicating that our approach is a viable method for identifying potential autophagy regulators. Only a single candidate made it past all of the screening criteria—Mot2p.

The Mot2/Not4 (modulator of transcription) protein was first characterized as a transcription factor that represses basal transcription of certain mating-specific genes, but was postulated to have a more global effect on gene expression (Cade and Errede 1994). Mot2p is also a phosphoprotein with five identified phosphorylation sites–Ser92, Ser312, Thr334, Ser342, and Thr 543 (Lau, Mulder et al. 2010) ( Figure 1B ). The Mot2 protein has a RING domain ( Figure 1B ) and functions as an E3 ligase to modulate cell stress responses (Albert, Hanzawa et al. 2002, Mulder, Inagaki et al. 2007). Mot2p was later identified as a subunit of the yeast Ccr4-Not complex (Liu, Badarinarayana et al. 1998). The Ccr4-Not complex regulates transcription (Liu, Badarinarayana et al. 1998, Badarinarayana, Chiang et al. 2000, Denis, Chiang et al. 2001), mRNA turnover (Badarinarayana, Chiang et al. 2000), and translational repression (Preissler, Reuther et al. 2015). Yin and colleagues investigated Ccr4-Not complex members Ccr4p and Pop2p, demonstrating bidirectional roles in autophagy regulation (Yin, Zhang et al. 2023). However, to the best of our knowledge, no one has examined the role of Mot2p in autophagy.

Nitrogen starvation is a robust trigger for autophagy in yeast (Delorme-Axford, Guimaraes et al. 2015). Autophagy-deficient cells display reduced viability under prolonged nitrogen starvation conditions (Tsukada and Ohsumi 1993, Bernard, Jin et al. 2015). Excessive autophagy results in a similar phenotype (Hu, McQuiston et al. 2015, Delorme-Axford, Wen et al. 2023). To examine the importance of the Mot2 protein under prolonged nitrogen starvation condition, we monitored the cell survival phenotype of cells lacking *MOT2* ( Figure 1C ). Loss of *MOT2* decreased cell survival under nutrient-rich conditions ( Figure 1C ), consistent with what has been observed by others (Lau, Mulder et al. 2010, Preissler, Reuther et al. 2015). Additionally, *mot2* Δ cells do not survive prolonged nitrogen starvation (7 days; Figure 1C ), suggesting that extended starvation in the absence of *MOT2* is detrimental.

Positive regulators are typically upregulated in response to autophagy inducing conditions (Xie, Nair et al. 2008, Yao, Delorme-Axford et al. 2015). Conversely, negative regulators are typically inactivated following autophagy (He and Klionsky 2009). To determine how nitrogen starvation impacts Mot2 protein levels, *MOT2* was chromosomally tagged at its C terminus with the hemagglutinin (3HA) epitope. Endogenous Mot2-3HA fusion protein levels were assessed by a time course of nitrogen starvation (0, 1, and 2 h) and examined by western blot analysis ( Figure 1D, E). Mot2-3HA fusion protein levels significantly decreased with nitrogen starvation in WT cells (>35% by 2 h; Figure 1D, E). In contrast, when cells are starved for nitrogen, *MOT2* mRNA levels are upregulated (~2-fold) in wild-type (WT) cells ( Figure 1F ), suggesting that *MOT2* /Mot2p may be differentially regulated under autophagy-inducing conditions. Notably, we observed a similar phenomenon with the yeast metabolic transcription factor Stb5p (Delorme-Axford, Wen et al. 2023). As a control, we also examined *UBC6* (a gene with no known connection to autophagy) expression levels during nitrogen starvation; no significant differences in *UBC6* levels were noted ( Figure 1F ).

During autophagy induction in yeast, *ATG8* levels increase (Bartholomew, Suzuki et al. 2012). Our YEASTRACT screen identified *ATG8* as a predicted target of Mot2p; there are multiple consensus sites along the *ATG8* promoter that lie within regions -302 to -163 upstream of the ATG +1 start site. Therefore, we investigated whether Mot2p could modulate *ATG8* mRNA expression ( Figure 1G ). During nutrient-rich conditions, *ATG8* levels were significantly higher (~2-fold) in *mot2* Δ cells compared to WT ( Figure 1G ). As expected, we observed that *ATG8* levels were upregulated when WT cells were starved for nitrogen (~6-fold; Figure 1G ). In *mot2* Δ cells, *ATG8* levels were enhanced (~9-fold) when cells were starved for nitrogen compared to nutrient-rich conditions (~2-fold; Figure 1G ). When cells were starved for nitrogen, we noted that *ATG8* levels were elevated in the *mot2* Δ strain compared to WT, although the difference was not statistically significant ( Figure 1G ).

Atg8 protein exists as two species in the cell – a non-lipidated soluble form and a lipidated phosphatidylethanolamine (PE)-conjugated species (Cheong and Klionsky 2008). Furthermore, Atg8 protein levels increase when autophagy is induced (Huang, Scott et al. 2000, Xie, Nair et al. 2008). Thus, assessing the total amount of Atg8 protein and its lipidation status is an indicator of autophagy (Delorme-Axford, Abernathy et al. 2018). Given that we identified Mot2p as a potential regulator of *ATG* genes ( Figure 1A ), and cells lacking *MOT2* do not survive prolonged nitrogen starvation ( Figure 1C ), we examined whether loss of *MOT2* had an effect on autophagy and Atg8 protein levels in *mot2* Δ cells ( Figure 1H ). We found that cells lacking *MOT2* had an increased amount of Atg8 protein relative to the WT under nutrient-rich and starved conditions ( Figure 1H ), supporting the idea that Mot2p negatively regulates Atg8 protein levels, and potentially, autophagy.

Here we present data supporting a role for Mot2p as a regulator of cell survival, *ATG8* , and potentially, autophagy. These findings add to the repertoire of studies that have utilized YEASTRACT (Teixeira, Monteiro et al. 2006) to identify regulators of *ATG* genes and autophagy (Yao, Delorme-Axford et al. 2015, Delorme-Axford, Abernathy et al. 2018, Delorme-Axford, Wen et al. 2023). These results serve as a basis for future work aimed at elucidating the role of Mot2p in the regulation of *ATG8* /Atg8p and autophagy.

## Methods

***Yeast Strains, Media, and Cell Culture:*** Yeast cells were grown in YPD (1% yeast extract, 2% peptone, and 2% glucose) medium from Gibco (A1374501). To induce autophagy, cells were grown to mid-log phase in YPD, then shifted to nitrogen starvation medium (SD-N; 0.17% yeast nitrogen base without ammonium sulfate or amino acids and 2% glucose) for the specified time points. Chromosome tagging with 3HA was performed using established methods (Longtine, McKenzie et al. 1998).

***Yeast Growth Assay:*** Yeast growth assays were performed as previously described (Yin, Zhang et al. 2023) with the following modifications. Yeast cells were cultured in YPD to mid-log phase and then shifted to SD-N for the time points indicated. An aliquot of cells (1 OD _600_ unit) was removed from each culture and serially diluted. Each dilution (5 μl) was spotted on YPD plates. Cells were grown at 30°C for 2 days before being imaged with a ChemiDocTouch imaging system (Bio-Rad).

***RNA and Real-Time Quantitative PCR (RT-qPCR):*** Yeast cells were cultured in YPD to mid-log phase and then shifted to SD-N (1 h) for autophagy induction. Cells (1 OD _600_ unit) were collected at the indicated time point, and the pellets were frozen in liquid nitrogen. Total RNA was extracted using the NucleoSpin RNA extraction kit (Clontech, 740955.250). Reverse transcription was carried out using the High-Capacity cDNA Reverse Transcription Kit (Applied Biosystems/Thermo Fisher Scientific, 4368814). For each sample, 1 µg RNA was used for cDNA synthesis. RT-qPCR was performed using the Power SYBR Green PCR Master Mix (Applied Biosystems/Thermo Fisher Scientific, 4367659) in a CFX Opus 96 (Bio-Rad, 12011319) real-time PCR machine. For all RT-qPCR experiments, melt curves were run after the PCR cycles to verify primer specificity. Relative gene expression was calculated using the 2 ^−ΔΔCT^ method (Livak and Schmittgen 2001), normalized to *SLD3* levels.

***SDS-PAGE and Western Blots:*** SDS-PAGE and western blots were performed as previously described (Cheong and Klionsky 2008, Delorme-Axford, Tasmi et al. 2023). Western blots were visualized using an Azure 600 (Azure Biosystems) or an iBright (Thermo Fisher Scientific) imaging system. Densitometry for western blots was performed using ImageJ ( https://imagej.net/ij/ ).

***Statistical analysis:*** The two-tailed unpaired *t* test was used to determine statistical significance with GraphPad Prism (GraphPad Software, USA). For Figure 1, *p* values are as follows: * *p* <0.05; ** *p* <0.01; *** *p* <0.001; NS indicates not significant. A *p* value < 0.05 was considered significant.

## Reagents

**Table:** 

***Saccharomyces cerevisiae* strains used in this study are as follows: **		
**Name**	**Genotype**	**Reference**
BY4742	MATα *his3* ∆ *1 leu2* ∆ *0 lys2* ∆ *0 ura3* ∆ *0*	Horizon Discovery
EWA018	SEY6210, *MOT2-3HA::TRP1*	This study
*mot2* ∆	BY4742, *mot2* ∆ *::KANMX*	Horizon Discovery
YAB369	YTS158, *atg8* Δ:: *HIS5*	(Delorme-Axford, Abernathy et al. 2018)
YTS158	BY4742, *pho13* Δ *::KANMX pho8::pho8* Δ *60*	(He, Song et al. 2006)
SEY6210	MAT *α his3∆200 leu2-3,112 lys2-801 suc2-∆9 trp1∆901 ura3-52*	(Robinson, Klionsky et al. 1988)
**RT-qPCR primer sequences used in this study are as follows:**		
**Name**	**Sequence (5' – 3')**	**Reference**
*ATG8-F*	GAAGGCCATCTTCATTTTTGTC	(Bernard, Jin et al. 2015)
*ATG8-R*	TTCTCCTGAGTAAGTGACATAC	(Bernard, Jin et al. 2015)
*MOT2-F*	ACGAAAACTCCCACCCAACC	This study
*MOT2-R*	CAAAGATACCCGGTGGAGGG	This study
*SLD3-F*	CGCAACTTCAAAGCATCATTGAATCGC	(Bernard, Jin et al. 2015)
*SLD3-R*	GGGGCTTATTAGTGGGAGTAGAGG	(Bernard, Jin et al. 2015)
*UBC6-F*	GATACTTGGAATCCTGGCTGGTCTGTCTC	(Teste, Duquenne et al. 2009)
*UBC6-R*	AAAGGGTCTTCTGTTTCATCACCTGTATTTGC	(Teste, Duquenne et al. 2009)
**Antibodies used in this study are as follows:**		
**Name**	**Identifier (Source)**	**Concentration**
mouse monoclonal anti-Atg8 (G-10)	Cat# sc-373963 (Santa Cruz Biotechnology)	1:1,000
mouse monoclonal anti-GAPDH (1E6D9)	Cat# 60004-1-Ig (Proteintech)	1:20,000
rabbit polyclonal anti-HA tag	Cat# 51064-2-AP (Proteintech)	1:10,000
mouse monoclonal anti-Pgk1 (22C5D8)	Cat# 459250 (Invitrogen)	1:5,000
